# Pupillary responses to the glare illusion in normal pressure hydrocephalus: insights into network dysfunction and neurodegenerative comorbidities

**DOI:** 10.1007/s10072-026-09015-2

**Published:** 2026-04-09

**Authors:** Ai Kawamura, Yuya Kinzuka, Nobuko Kawakami, Keisuke Morihara, Kazuo Kakinuma, Kazuto Katsuse, Shiho Matsubara, Chifumi Iseki, Shigenori Kanno, Shigeki Nakauchi, Kyoko Suzuki

**Affiliations:** 1 Department of Behavioral Neurology and Cognitive Neuroscience, Tohoku University Graduate School of Medicine, Sendai, Japan; 2 Department of Computer Science and Engineering, Toyohashi University of Technology, Toyohashi, Japan; 3 Department of Neurology and Stroke Medicine, Yokohama City University Graduate School of Medicine, Yokohama, Japan; 4 Department of Neurology, Graduate School of Medicine, The University of Tokyo, Tokyo, Japan

**Keywords:** Pupillometry, Glare illusion, Normal pressure hydrocephalus, Visual perception, Neurodegenerative disease, Lewy body disease

## Abstract

**Background:**

The glare illusion, in which a central area appears self-luminous due to surrounding luminance gradients, induces pupillary constriction, suggesting involvement of higher-order visual processing beyond the brainstem-mediated light reflex. However, its neural underpinnings remain unclear. This study investigated pupillary responses to glare and control stimuli in patients with normal pressure hydrocephalus (NPH), characterized by predominant white matter pathology, and healthy controls. We also examined whether pupillary responses might be influenced by potential comorbid neurodegenerative conditions.

**Methods:**

Pupillary responses were recorded using pupillometry while participants viewed luminance-matched glare and control stimuli. Pupillary constriction amplitude was evaluated for each condition and compared between conditions. Subgroup analyses were performed according to dopamine transporter (DAT) uptake status.

**Results:**

Patients with NPH showed generally attenuated stimulus-evoked pupillary constriction amplitude compared with healthy controls. However, in the overall NPH group, pupillary constriction in response to glare stimuli remained greater than that to control stimuli. This condition-dependent difference was consistently observed for constriction amplitude, but not for constriction velocity. In subgroup analyses, patients with reduced DAT uptake showed no clear difference between conditions, whereas those with preserved DAT uptake retained greater constriction to glare stimuli.

**Conclusions:**

Pupillary constriction may provide a potential physiological marker of higher-order visual processing. The overall attenuation of pupillary responsiveness in NPH is consistent with altered large-scale network regulation associated with white matter pathology. Given the small sample size, the absence of a condition-dependent difference in patients with reduced DAT uptake should be interpreted with caution and warrants further investigation.

**Supplementary Information:**

The online version contains supplementary material available at 10.1007/s10072-026-09015-2.

## Introduction

Pupillary responses have gained attention as biomarkers for neurodegenerative disorders such as Alzheimer’s disease and Parkinson’s disease. The pupil light reflex (PLR), which refers to pupil constriction in response to light and dilation in darkness, is a physiological measure linked to disease-related neural changes. In these disorders, altered PLR has been linked to neurotransmitter and autonomic dysfunction as well as retinal degeneration [1, 2]. Because these diseases involve neurotransmitter abnormalities, pupillary responses may reflect both cognitive and physiological changes.

Normal pressure hydrocephalus (NPH) is a type of dementia characterized by gait disturbances, urinary incontinence, and cognitive impairment. Unlike Alzheimer’s disease and Parkinson’s disease, NPH does not primarily involve neurotransmitter deficits, but rather widespread white matter damage resulting from ventricular enlargement and cerebrospinal fluid (CSF) dysregulation [3, 4]. NPH mainly affects older adults and may coexist with neurodegenerative diseases. Although not typically associated with neurotransmitter dysfunction, some NPH patients show dopamine transporter (DAT) abnormalities, suggesting comorbidities with Parkinson’s disease or dementia with Lewy bodies, collectively referred to as Lewy body disease [5]. Comorbid Lewy body disease produces symptoms overlapping with NPH, complicating diagnosis. Notably, Lewy body disease has been linked to distinctive impairments in visual perception, including a reduced susceptibility to visual illusions [6, 7]. These findings suggest that objectively assessed visual illusion paradigms may be sensitive to perceptual deficits associated with neurodegenerative comorbidities, offering a potential tool for identifying such conditions in patients with NPH.

PLR is primarily governed by the autonomic nervous system. Although it is traditionally regarded as a reflexive response to light intensity, recent evidence suggests that PLR is also influenced by higher-order cognitive factors, such as attention, semantic associations, and expectations [8, 9]. A promising way to examine these cognitive influences is through visual illusions—particularly the glare illusion [10], which creates the vivid impression of central brightness or self-luminescence by surrounding a white region with a luminance gradient. This illusion increases perceived brightness by approximately 30% [11] and elicits stronger pupillary constriction compared to control stimuli with matched luminance. These effects suggest involvement of cortical mechanisms in brightness estimation and visual integration. [12–15]. Furthermore, perceptual brightness has been shown to modulate not only pupil size but also temporal perception [16].

Identifying comorbid neurodegenerative diseases in patients with NPH remains a significant clinical challenge, owing to overlapping symptomatology and invasive diagnostics, which are often costly and burdensome. This underscores the need for non-invasive, physiological markers to detect comorbidities. While visual illusions have been employed in prior studies to explore perceptual disturbances in neurological disorders, these investigations have typically relied on subjective self-reports [6, 7, 17]. However, subjective measures are susceptible to cognitive and perceptual biases, particularly in clinical populations. In contrast, pupillometry provides an objective and involuntary measure of perceptual processing by capturing automatic pupil responses to visual stimuli, independent of active participant input. As such, pupillometry may be especially well-suited for detecting subtle perceptual alterations linked to comorbid neurodegeneration in NPH.

In addition to its clinical utility, glare-induced pupillary responses may also provide insight into the neural mechanisms underlying perceptual illusions. NPH offers a unique model in this regard: while it involves widespread white matter damage disrupting large-scale brain networks, the primary visual and neurotransmitter systems remain relatively intact. Pupil dynamics reflect both subcortical autonomic control and activity in distributed cortical networks involved in cognitive and perceptual processing [18]. Thus, altered pupillary responses in NPH may indicate impaired network efficiency independent of neurotransmitter dysfunction. Studying how white matter pathology in NPH affects glare illusion processing could help disentangle the relative contributions of cortical network integrity and neuromodulatory systems to illusion-induced pupillary responses.

The aim of this study was to examine whether the glare illusion effect is preserved in normal pressure hydrocephalus (NPH), a disorder characterized by predominant white matter pathology, and whether it may be influenced by comorbid neurodegenerative disease. We objectively assessed pupillary constriction to glare stimuli in NPH patients and compared responses between those with and without reduced DAT uptake. This approach may help evaluate the potential utility of pupillometry as a non-invasive physiological measure related to comorbid neurodegenerative disease in NPH. In doing so, it may also provide insights into the neural mechanisms underlying illusion-induced pupillary responses.

## Methods

### Subjects

This study enrolled 45 patients (18 women, 27 men) diagnosed with NPH at the Tohoku University Hospital between February 2022 and December 2024. NPH diagnosis was confirmed by board-certified neurologists based on the following criteria: (1) aged 60 years or older, (2) at least one main symptom (gait disturbance, dementia, and urinary incontinence), (3) MRI evidence of ventricular enlargement, defined as an Evans index > 0.3, (4) cerebrospinal fluid pressure < 200 mmH₂O with normal cell count and protein levels, (5) absence of other conditions that could explain the symptoms, and (6) no history of diseases known to cause ventricular dilation. Among the 45 patients, 39 were diagnosed with idiopathic NPH (iNPH), including 25 pre- and 14 postoperative cases. Additionally, four patients were diagnosed with secondary NPH (sNPH), and two with panventriculomegaly, characterized by a wide foramen of Magendie and a large cisterna magna (PaVM).

The study also included a group of 38 healthy controls (HCs), including 16 women and 22 men. Inclusion criteria for HCs were: (1) aged 70 years or older, (2) no history of neurological disorders, and (3) no ocular diseases other than mild cataracts, with sufficient visual acuity for daily activities.

### Clinical assessments

The standard neuropsychological examinations assessing general cognitive functions included the Mini-Mental State Examination (MMSE) [19] and the Japanese version of the Montreal Cognitive Assessment (MoCA-J) [20]. The severity of the disease in NPH patients was evaluated using the idiopathic normal pressure hydrocephalus grading scale (iNPHGS) [21]. Near visual acuity was assessed at a distance of 40 cm using a near vision chart and recorded in M-units.

To examine the comorbidity of other diseases associated with gait disturbance, DAT imaging was performed in NPH patients using single-photon emission computed tomography (SPECT) with ^123^I–N-ω-fluoropropyl-2β-carboxymethoxy-3β-(4-iodophenyl)nortropane ([^123^I]FP-CIT). Based on the specific binding ratio (SBR), corrected using a CSF region mask, patients were classified into two groups—Normal-DAT and Low-DAT—according to the lower limit of the 95% predictive interval for each age group in healthy Japanese individuals, as reported by Matsuda et al. [22].

### Stimuli and apparatus

**Fig. 1:**
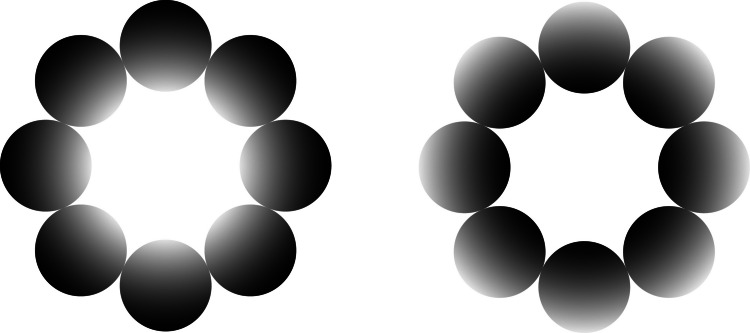
Glare and Control stimuli. The glare stimulus (left) features a circular pattern with increasing luminance toward the central white region, while the control stimulus (right) has the opposite gradient. The center of the glare illusion appears brighter. The luminance values shown here differ from those used in the study

Glare illusion and control stimuli with inverted luminance gradients were employed (Fig. 1). The Glare stimuli featured circular patterns with luminance increasing toward a central white region, while the control stimuli presented the same patterns rotated by 180°, resulting in a reversed gradient. This ensured that both stimulus types shared identical physical properties apart from gradient direction. Each stimulus set consisted of eight circular elements.

Chromaticity coordinates (xy) in the CIE 1931 color space were as follows: black (0.3127, 0.3290), blue (0.1653, 0.0921), and red (0.6132, 0.3413). The luminance gradients transitioned from these chromatic coordinates toward the achromatic point (0.3127, 0.3290). Across all colors, luminance ranged from 0.4116 cd/m^2^ to 80.91 cd/m^2^. The background and central region luminance were fixed at 40.52 cd/m^2^ and 80.91 cd/m^2^, respectively. Stimuli were displayed at a visual angle of 12.10°, with a centrally placed fixation cross (0.3° visual angle) used to support accurate pupil diameter measurement.The experiment was conducted in a dimly lit room (30 lx), with stimuli displayed using MATLAB R2024a (The MathWorks, Natick, MA, USA) and the Psychtoolbox-3 extension [23] on an LCD monitor (Display + +, Cambridge Research Systems Ltd, Rochester, UK; 1,920 × 1,080 pixels, 120 Hz refresh rate). Pupil responses were recorded from both eyes at a sampling rate of 500 Hz using an EyeLink Portable Duo eye-tracker (SR Research, Ottawa, Canada), positioned below the display to align with the participant’s line of sight. A chin rest stabilized the head at a 70 cm viewing distance.

### Procedure

**Fig. 2:**
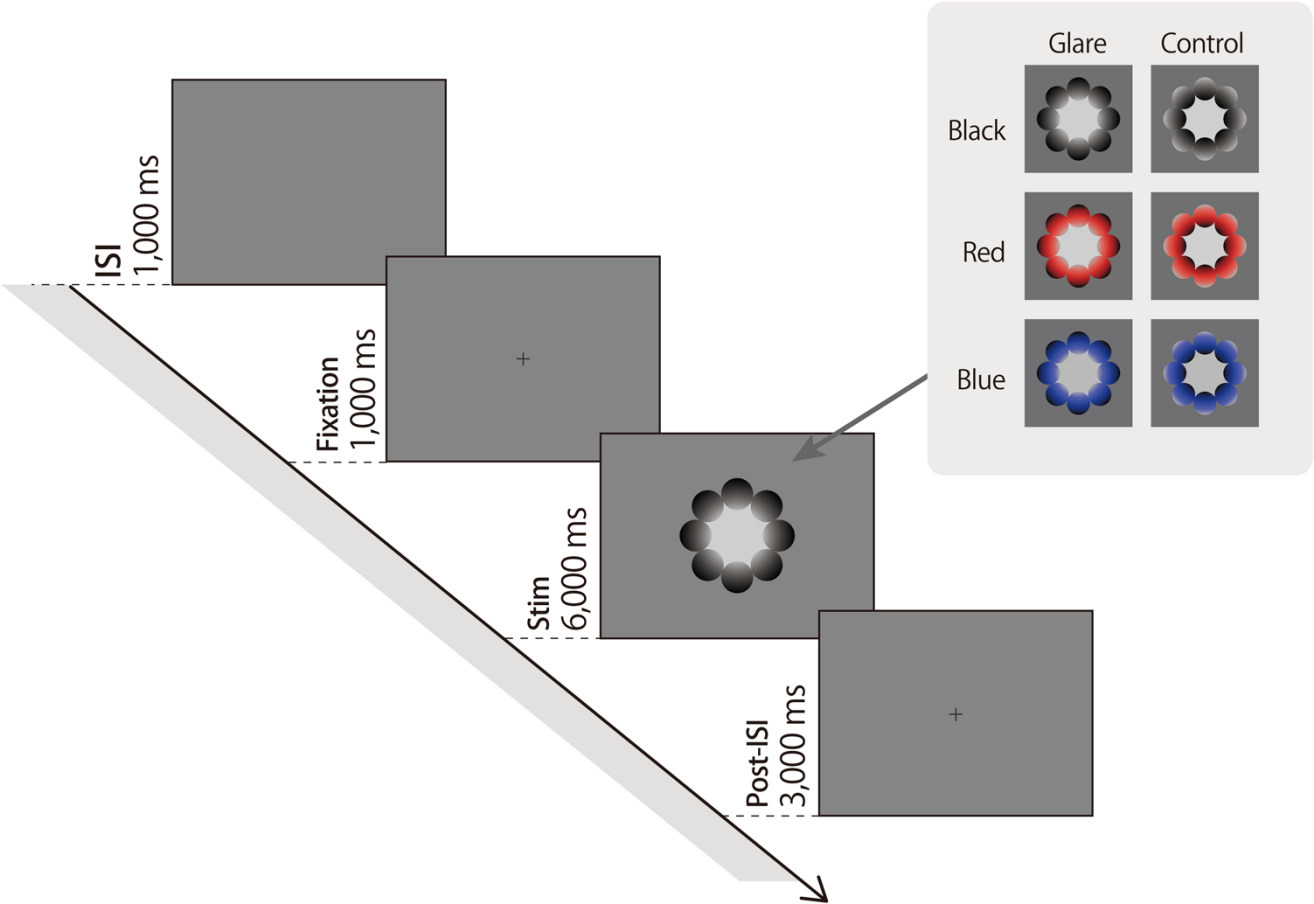
Experimental procedure. Experimental procedure including fixation, stimulus presentation, and measurement periods

Prior to each session, a standard five-point calibration was performed for the eye-tracker. Each trial consisted of a 1,000 ms inter-stimulus interval (ISI), a 1,000 ms fixation point (0.3-degree visual angle), 6,000 ms of Glare or Control stimuli, and a 3,000 ms fixation point (Fig. 2). The experiment was divided into three sessions, totaling 72 trials (~ 15 min). Six stimulus types (three colors × two gradient patterns; Glare and control stimuli) were randomly presented.

### Pupillary response analysis

Pupil data affected by blinks were interpolated using cubic-spline interpolation [24] in Python 3.9. We excluded trials containing artifacts, identified by excessive pupil response velocity changes. Additionally, two patients with NPH (one with iNPH and one with sNPH) whose trials were rejected in more than 50% of cases during pre-processing were excluded from the analysis due to insufficient data for reliable interpretation. Thus, the final sample consisted of 43 patients with NPH, including 38 with iNPH (24 preoperative and 14 postoperative), 3 with sNPH, and 2 with PaVM, along with 38 control participants. 

Pupil size, initially recorded in arbitrary units by the EyeLink system, was converted to millimeters using offline calibration. For time-course analysis, pupil diameter at stimulus onset was normalized to a pre-stimulus baseline. Maximum pupil constriction was defined as the minimum pupil diameter following stimulus onset, and maximum constriction velocity was calculated as the peak negative value of the first derivative of pupil diameter [1]. Mean values were computed separately for each stimulus color (black, blue, and red), as well as for the overall average across all colors. Baseline pupil diameter was defined as the mean absolute pupil size during the fixation period (from 50 ms before to the moment of stimulus onset).

### Statistical analysis

To assess disease-related effects, a two-way repeated measures ANOVA was performed on maximum pupil constriction and velocity, with stimulus condition (Glare, Control) as a within-subject factor and group (NPH, HC) as a between-subject factor. Prior to ANOVA, normality of the outcome variables was assessed using the Shapiro–Wilk test. Although some variables showed deviations from normality, ANOVA is considered robust to moderate violations of the normality assumption with balanced sample sizes. Demographic variables and baseline pupil diameter were compared using independent samples t-tests and chi-square tests, as appropriate. Bayesian t-tests were also conducted to calculate Bayes factors (BF₁₀), quantifying evidence for the null hypothesis regarding group differences in baseline pupil size. To investigate age-related effects, both Pearson’s correlation and a three-way ANOVA were employed, incorporating group, age (dichotomized by median split), and stimulus condition as factors.

To explore effects of DAT status, a two-way repeated measures ANOVA was performed within the NPH group, with stimulus condition (Glare, Control) and DAT status (Normal, Low) as factors. Statistical significance was set at *p* < 0.05, with multiple comparisons corrected using the Holm method. All analyses were conducted using JASP 0.19.3 (JASP Team) [25].

## Results

Table 1 presents the demographic characteristics of the subjects, excluding the two NPH patients with trial rejection. The age, MMSE and MoCA-J scores were significantly different between the NPH and HC groups. The severity of NPH, as indicated by iNPHGS, is shown in Table 1, and reflects a high proportion of clinically mild cases.

**Table 1:** Demographic characteristics of the final sample, excluding two NPH patients removed due to trial rejection

Variables	NPH (*n* = 43)	HC (*n* = 38)	*P* value
Age (years)	76.7 ± 4.9	73.7 ± 2.4	< 0.001
Sex (women/men)	18/25	16/22	0.982
Educational attainment (years)	12.7 ± 2.8	12.5 ± 1.9	0.685
Near visual acuity (M)	1.1 ± 0.4	1.2 ± 0.6	0.612
MMSE (/30)	24.0 ± 3.7	27.9 ± 1.9	< 0.001
MoCA-J (/30)	19.8 ± 3.8	24.0 ± 2.7	< 0.001
iNPHGS			
Gait	2.0 ± 0.6	-	-
Cognition	2.1 ± 0.8	-	-
Urination	1.2 ± 1.2	-	-
Total	5.2 ± 1.9	-	-

Data are presented as means ± SDs except for sex (women/men). The chi-square test (χ^2^ test) was used for gender, and the Student’s *t*-test was used for other comparisons

*HC* healthy controls; *iNPHGS* idiopathic normal pressure hydrocephalus grading scale; *MMSE* Mini-Mental State Examination; *MoCA-J* Japanese version of the Montreal Cognitive Assessment; *NPH* normal pressure hydrocephalus

For the maximum pupil constriction amplitude, significant main effects of stimulus pattern were observed for all colors [Black: F(1, 79) = 4.944, *p* = 0.029, *η*^2^*p* = 0.059; Blue: F(1, 79) = 10.809, *p* = 0.002, *η*^2^*p* = 0.120; Red: F(1, 79) = 6.466, *p* = 0.013, *η*^2^*p* = 0.076], as well as significant main effects of groups [Black: F(1, 79) = 9.146, *p* = 0.003, *η*^2^*p* = 0.104; Blue: F(1, 79) = 14.365, *p* < 0.001, *η*^2^*p* = 0.120; Red: F(1, 79) = 6.775, *p* = 0.011, *η*^2^*p* = 0.076]. No significant stimulus pattern × group interaction was observed for any color (Supplementary Fig. 1a). Across all colors, pupil constriction amplitude was significantly greater in response to Glare stimuli compared to Control stimuli, indicating the glare illusion effect. However, the NPH group exhibited smaller constriction amplitudes than the HC group.

**Fig. 3:**
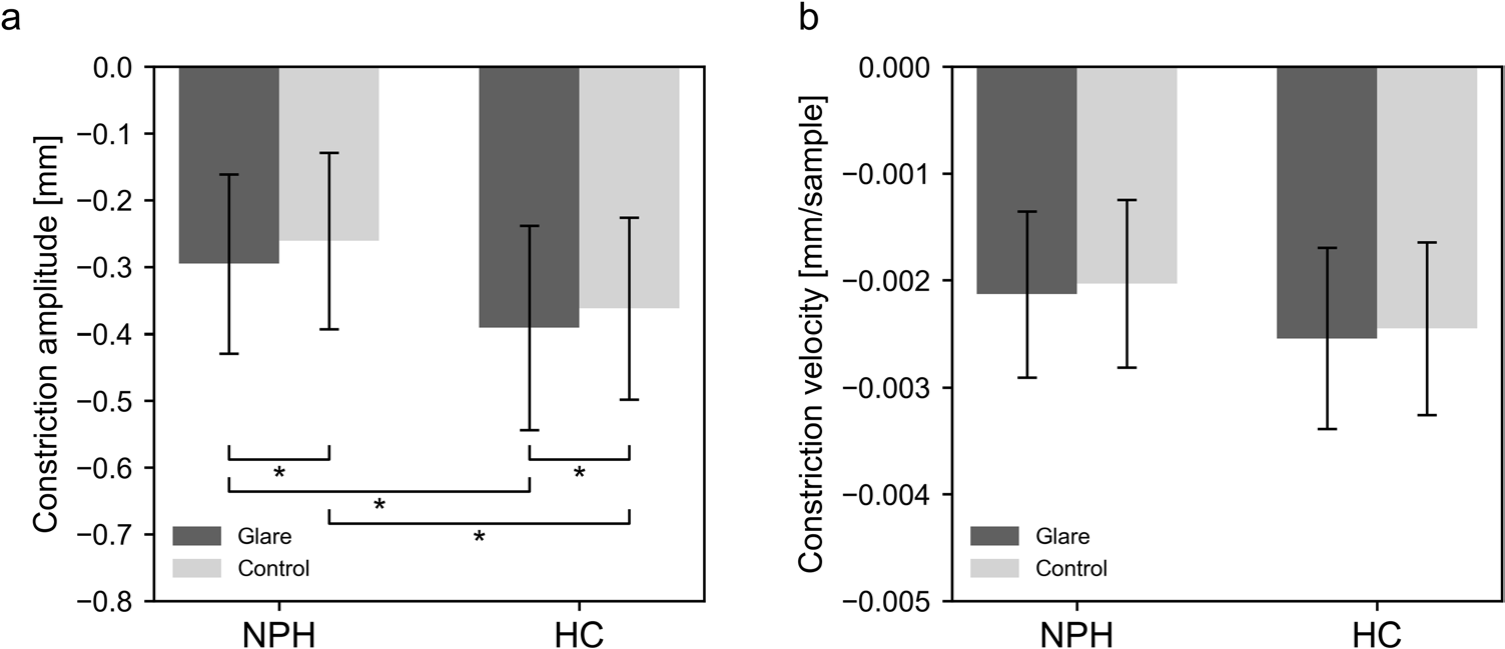
Pupillary response to glare and control stimuli across groups (all colors). Pupillary light reflex responses pooled across all three color conditions are shown as (**a**) constriction amplitude (maximum pupil constriction amplitude) and (**b**) constriction velocity (maximum pupil constriction velocity). Error bars indicate standard deviation. *Statistically significant differences (*p* <.05). HC = healthy controls; NPH = normal pressure hydrocephalus

Given the consistency of this trend across colors, a two-way repeated measures ANOVA was performed on the aggregated data. For the maximum pupil constriction amplitude, the analysis revealed significant main effects of both stimulus pattern (F(1, 79) = 17.447, *p* < 0.001, *η*^2^*p* = 0.181) and group (F(1, 79) = 10.836, *p* = 0.001, *η*^2^*p* = 0.121), with no significant interaction (F(1, 79) = 0.109, *p* = 0.743, *η*^2^*p* = 0.001) (Fig. 3a). Post-hoc analysis showed the NPH group exhibiting significantly smaller pupil constriction amplitude compared to the HC group for both Glare (*p* = 0.010) and Control stimuli (*p* = 0.005). In both groups, Glare stimuli induced greater constriction than Control stimuli (NPH: *p* = 0.006; HC: *p* = 0.020).

The maximum pupil constriction velocity was analyzed for both stimulus pattern and group across all colors (Supplementary Fig. 1b). A two-way repeated measures ANOVA revealed significant main effects of both stimulus pattern and group for the Blue stimuli [Pattern: F(1, 79) = 10.456, *p* = 0.002, *η*^2^*p* = 0.117; Group: F(1, 79) = 6.042, *p* = 0.016, *η*^2^*p* = 0.071], with no significant interaction [F(1, 79) = 1.786, *p* = 0.185, *η*^2^*p* = 0.022]. For the Red stimuli, a significant main effect of group was observed [F(1, 79) = 7.643, *p* = 0.007, *η*^2^*p* = 0.088]; however, no significant main effect of pattern or interaction was found [Pattern: F(1, 79) = 0.327, *p* = 0.569, *η*^2^*p* = 0.004; Interaction: F(1, 79) = 2.787, *p* = 0.099, *η*^2^*p* = 0.034]. For the Black stimuli, neither main effects nor interactions were significant [Pattern: F(1, 78) = 1.765, *p* = 0.188, *η*^2^*p* = 0.022; Group: F(1, 78) = 2.244, *p* = 0.138, *η*^2^*p* = 0.028; Interaction: F(1, 78) = 0.195, *p* = 0.660, *η*^2^*p* = 0.002]. Across all colors, the NPH group exhibited a slower maximum pupil constriction velocity compared to the HC group.

A separate two-way repeated measures ANOVA on maximum pupil constriction velocity across all colors revealed significant main effects of both stimulus pattern and group [Pattern: F(1, 79) = 5.614, *p* = 0.020, *η*^2^*p* = 0.066; Group: F(1, 79) = 5.582, *p* = 0.021, *η*^2^*p* = 0.066], with no significant interaction [F(1, 79) = 0.015, *p* = 0.904, *η*^2^*p* = 1.866 × 10⁻^4^] (Fig. 3b). However, post-hoc comparisons did not reveal any significant pairwise differences.

Baseline pupil diameter did not significantly differ between the two groups (NPH: NPH: 7.2 ± 0.8 mm, HC: 7.1 ± 0.8 mm, *p* = 0.617). Bayesian analysis indicated a Bayes factor (BF₁₀) of 0.258 for baseline pupil diameter, providing moderate evidence supporting the null hypothesis [26, 27], suggesting no difference in baseline pupil diameter between the groups.

Because the NPH group had significantly older average age than the HC group, additional analyses were performed to evaluate the potential influence of age on pupil responses. Pearson’s correlation analyses revealed no significant associations between age and maximum pupil constriction amplitude (Glare: *p* = 0.877, Control: *p* = 0.889) or maximum pupil constriction velocity (Glare: *p* = 0.478, Control: *p* = 0.390). In the three-way repeated ANOVA measures, significant main effects of stimulus pattern [F(1, 77) = 17.442, *p* < 0.001, *η*^2^*p* = 0.185] and group [F(1, 77) = 12.969, *p* < 0.001, *η*^2^*p* = 0.144] were observed for maximum pupil constriction amplitude. However, neither the main effect of age [F(1, 77) = 1.603, *p* = 0.209, *η*^2^*p* = 0.020] nor any other interactions involving age were significant. Similarly, for maximum pupil constriction velocity, significant main effects of stimulus pattern [F(1, 77) = 5.299, *p* = 0.024, *η*^2^*p* = 0.064] and group [F(1, 77) = 6.440, *p* = 0.013, *η*^2^*p* = 0.077] were found. However, the main effect of age [F(1, 77) = 1.228, *p* = 0.271, *η*^2^*p* = 0.016], as well as all other interactions involving age, were non-significant.

**Fig. 4:**
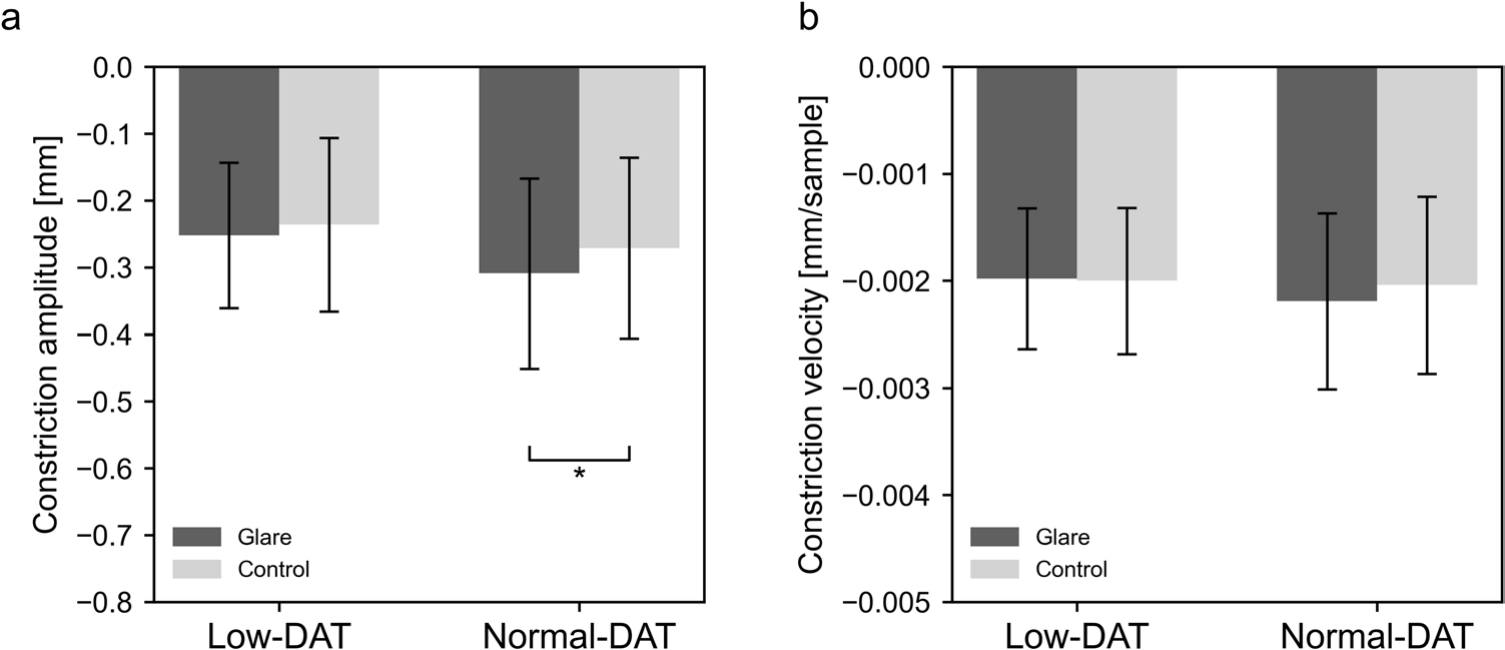
Pupillary responses in NPH patients stratified by DAT uptake status (Normal vs. Low). Pupillary responses pooled across the three color conditions are shown as (**a**) constriction amplitude and (**b**) constriction velocity. Error bars indicate standard deviation. *Statistically significant differences (*p* <.05). DAT = dopamine transporter; NPH = normal pressure hydrocephalus

To explore the effect of comorbid neurodegenerative diseases, participants were divided based on DAT uptake into a group with normal accumulation (Normal-DAT, *n* = 31) and one with reduced accumulation (Low-DAT, *n* = 11). A two-way repeated ANOVA measures revealed a significant main effect of stimulus pattern on maximum pupil constriction amplitude [F(1, 40) = 4.421, *p* = 0.042, *η*^2^*p* = 0.100], reporting no significant main effect of group or interaction [F(1, 40) = 1.025, *p* = 0.317, *η*^2^*p* = 0.025; F(1, 40) = 0.719, *p* = 0.401, *η*^2^*p* = 0.018] (Fig. 4a). Post-hoc tests showed a significant simple main effect of stimulus pattern in the Normal-DAT group (*p* = 0.012) but not in the Low-DAT group (*p* = 0.347). For maximum pupil constriction velocity, no significant main effects of stimulus pattern [F(1, 40) = 0.853, *p* = 0.361, *η*^2^*p* = 0.021] or group [F(1, 40) = 0.204, *p* = 0.654, *η*^2^*p* = 0.005], nor any significant interaction [F(1, 40) = 1.869, *p* = 0.179, *η*^2^*p* = 0.045], were found (Fig. 4b).

To further illustrate illusion-specific modulation independent of overall pupillary response magnitude, an Illusion Effect Index (IEI; Glare − Control) was calculated. No significant differences were observed between healthy controls and NPH patients or between DAT subgroups (Supplementary Table 1, 2).

## Discussion

This is the first study to investigate pupillary responses to the glare illusion in a clinical population. Our findings demonstrate that NPH patients exhibit preserved but attenuated pupillary constriction amplitude in response to the glare illusion. While this illusion-related effect was robust for constriction amplitude, velocity measures were less consistent across stimulus conditions. Furthermore, the illusion effect was diminished in patients with reduced DAT uptake, suggesting a modulatory influence of the comorbid neurodegenerative pathology. These results support the potential clinical utility of pupillometry as a non-invasive physiological marker for detecting underlying disease mechanisms in NPH.

### General attenuation of pupillary responses in NPH

NPH patients showed preserved but attenuated pupillary responses to the glare illusion, with reductions in constriction amplitude across stimulus types. Constriction velocity showed a similar overall tendency but was more variable, particularly under certain color conditions. This general reduction likely reflects impaired modulation of pupil dynamics, consistent with the known white matter pathology in NPH.

Pupil size is regulated not only by autonomic reflexes but also by higher-order networks involved in attention, salience detection, and executive function. Recent fMRI studies incorporating pupillometry have shown that pupil dynamics are closely linked to large-scale brain networks, extending beyond autonomic control solely driven by luminance [18]. Notably, pupil diameter shows a negative correlation with activity in sensory and visual cortices, indicating a role in modulating sensory processing beyond simple light adaptation—potentially via neuromodulatory mechanisms [28, 29]. Conversely, the rate of pupil diameter change positively correlates with activity in brain regions implicated in salience detection, error monitoring, and decision-making, including the locus coeruleus, thalamus, posterior cingulate cortex, dorsal anterior cingulate and paracingulate cortices, orbitofrontal cortex, and right anterior insular cortex [18]. These findings underscore that pupil responses reflect dynamic, integrative neural processes rather than purely autonomic reflexes.

Given the strong relationship between pupillary responses and large-scale brain networks, it is plausible that the attenuation of pupillary responses in NPH is a consequence of network inefficiency resulting from white matter damage. The pathophysiology of NPH is primarily linked to impaired CSF absorption, leading to ventricular enlargement. Large ventricles in turn exert mechanical compression on periventricular white matter, causing ischemic damage to white matter axons [3, 4]. Structural connectivity analyses in NPH have revealed consistent white matter alterations in the periventricular region, as well as the frontal, temporal, and parietal lobes [30]. Moreover, clinical improvements following shunt surgery have been associated with partial recovery of white matter integrity, highlighting the role of its pathology in the clinical symptoms of NPH [31].

Importantly, our findings show no significant difference in baseline pupil diameter between NPH and control groups, suggesting that tonic activity of the locus coeruleus remains preserved in NPH. Instead, the observed reduction in stimulus-evoked pupillary responses likely reflects impaired top-down modulation due to network inefficiency, rather than intrinsic dysfunction of the locus coeruleus.

These interpretations are consistent with findings that white matter integrity is critical for pupil regulation [18, 32]. Additionally, NPH has been associated with reduced activity in the default mode network, a key network involved in cognitive control and self-referential processing [33, 34]. Given that the posterior cingulate cortex serves as a hub for both the DMN and pupillary control networks [18], its dysfunction in NPH may hinder coordination between the cortical and subcortical structures, potentially affecting pupillary responses.

The more pronounced effects observed in the pooled analyses may partly reflect an improved signal-to-noise ratio obtained by averaging across color conditions, as responses to individual colors exhibit greater variability. Notably, substantial inter-individual variability was observed in pupillary responses across both healthy controls and NPH patients. Such variability is characteristic of pupillary dynamics, which are influenced by individual differences in baseline autonomic tone, cognitive state, and aging. Moreover, NPH itself represents a clinically heterogeneous condition with variable degrees of white matter involvement and comorbid pathology. Therefore, the observed variability likely reflects a combination of inherent physiological differences in pupillary dynamics and disease-related heterogeneity within the NPH population. Importantly, despite this variability, statistically significant group differences were still observed, suggesting that the underlying effect of altered pupillary modulation in NPH is robust at the group level.

### Attenuated glare illusion–related pupillary modulation in NPH patients with reduced DAT uptake: exploratory implications for comorbid neurodegeneration

The present findings suggest that NPH patients with reduced DAT uptake may exhibit attenuated pupillary modulation in response to glare illusion stimuli compared with those with preserved DAT uptake. Given the limited sample size and substantial inter-individual variability within this subgroup, these findings should be interpreted as exploratory and hypothesis-generating.

One potential contributing factor may involve cholinergic dysfunction, which plays a key role in pupillary control and is prominently affected in disorders such as dementia with Lewy bodies. Disruption of cholinergic modulation could attenuate pupillary constriction in response to illusion-induced brightness. However, the relative preservation of responses to control stimuli suggests that the observed reduction in glare-related modulation is unlikely to reflect global autonomic impairment alone, and may instead involve altered cortical or perceptual modulation of pupillary dynamics.

Another possible explanation is impaired higher-order visual integration associated with neurodegenerative changes reflected by reduced DAT uptake. Reduced DAT uptake itself should not be interpreted as directly causing altered illusion-related visual processing. Rather, DAT reduction may reflect comorbid Lewy body pathology, which could influence visual integration processes and their coupling to pupillary responses. Although direct links between dopaminergic dysfunction and glare illusion processing have not been established, prior studies in Lewy body disease provide relevant context. For example, delayed recognition of illusory contours in Parkinson’s disease has been associated with hypometabolism in the lateral occipital complex [6], and marked deficits in illusion perception have been reported in dementia with Lewy bodies [7], suggesting disruption of higher-order visual networks.

The present paradigm assesses pupillary modulation rather than subjective perceptual experience, and therefore cannot determine whether the attenuation reflects reduced illusion perception itself or altered responsiveness to perceptual processing. Moreover, although prior studies indicate that glare illusion effects arise from early visual processing and are shaped by cognitive and contextual influences [35–38], the anatomical and neurochemical substrates remain incompletely understood.

### Clinical implications and future directions

Our findings highlight potential clinical implications. First, the overall reduction in pupillary responses in NPH suggests that pupillometry may serve as a non-invasive tool to monitor disease progression and treatment effects. Post-surgical pupillary changes may reflect recovery of white matter and function. Second, glare illusion responses may provide indirect insight into possible neurodegenerative comorbidity in NPH patients. The association between reduced DAT uptake and diminished pupillary modulation raises the possibility that altered glare illusion–related pupillary responses may reflect comorbid Lewy body pathology in some NPH patients, although this remains speculative.

Future studies combining pupillometry, visual psychophysics, and neuroimaging will be important for clarifying the neural mechanisms underlying glare illusion–related pupillary responses and their clinical significance in NPH. Multimodal approaches incorporating structural and functional imaging may also help determine how white matter pathology interacts with neurodegenerative processes to influence visual–autonomic coupling. In addition, longitudinal studies should examine whether these pupillary alterations correlate with neurodegenerative progression, disease burden, or responsiveness to therapeutic interventions, such as shunt surgery for NPH. Finally, future work may also explore monocular pupillary responses to evaluate potential laterality effects, which may provide additional insights in neurodegenerative conditions with asymmetric pathology.

### Limitations

This study offers novel insights but has several limitations. First, although our sample size was adequate for statistical analysis, it is relatively small, particularly in the subgroup of NPH patients with reduced DAT uptake. Larger, more diverse cohorts are needed to validate our findings and explore heterogeneity in NPH. Second, the NPH group had significantly older average age than the control group, suggesting that age-related changes may have influenced the results. Although our three-way ANOVA found no significant interaction between age and pupil responses, prior studies have shown that pupil size decreases with age [39] and that aging affects dynamic pupillary responses, including constriction amplitude and velocity [40]. However, our results primarily reflect reductions in stimulus-evoked responses rather than baseline diameter, indicating that age alone is unlikely to account for the observed effects. Instead, impaired neural regulation—possibly linked to white matter damage—may be the more plausible driver. Third, the present study did not include direct measures of white matter integrity or network connectivity, and therefore mechanistic interpretations regarding network inefficiency remain speculative. The cross-sectional design further limits causal inference regarding the relationship between white matter pathology and altered pupillary responses to the glare illusion. Future longitudinal studies incorporating diffusion imaging and connectivity analysis are warranted to clarify these mechanisms. Finally, although luminance was carefully controlled, subtle color-specific differences in spectral sensitivity may have contributed to variability in velocity measures, particularly under red stimulus conditions. One previous study has suggested that the effect of the glare illusion may vary with stimulus color, reporting stronger effects for blue stimuli [15]. However, this limited evidence does not readily explain the divergent pattern observed for the red stimulus in the present study, and the influence of color-dependent spectral sensitivity on illusion-related pupillary dynamics therefore remains uncertain.

## Conclusion

This study is the first to investigate pupillary responses to the glare illusion in patients with neurological disorders. NPH patients exhibited significantly attenuated pupillary constriction amplitude to both glare and control stimuli, suggesting altered stimulus-evoked pupillary modulation. These findings are consistent with disrupted large-scale network regulation, potentially associated white matter pathology in NPH. Within the NPH group, reduced glare illusion–related modulation was observed in patients with decreased DAT uptake. Given the limited sample size, this finding should be interpreted as exploratory and may reflect comorbid neurodegenerative pathology rather than a direct dopaminergic effect. Together, these results suggest that pupillometry may provide a useful, non-invasive physiological index of altered network regulation in NPH and highlight its potential value for investigating possible neurodegenerative comorbidity in future studies.

## Supplementary Information

Below is the link to the electronic supplementary material.

Supplementary file1 (DOCX 17 KB)

Supplementary file2 (DOCX 17 KB)

Supplementary file3 Supplementary Figure 1. Pupillary responses to glare and control stimuli across groups for each color condition and DAT status. Pupillary responses analyzed separately for each color condition (black, blue, and red) are shown as (a) constriction amplitude and (b) constriction velocity. Corresponding responses stratified by DAT uptake status within the NPH group (Normal-DAT vs. Low-DAT) are shown in (c) constriction amplitude and (d) constriction velocity. Black, blue, and red conditions are displayed from left to right in each row. Error bars indicate standard deviation. *Statistically significant differences (*p* <.05). HC = healthy controls; NPH = normal pressure hydrocephalus; DAT = dopamine transporter. (TIFF 206465 KB)

## Data Availability

Anonymized data supporting the findings of this study will be shared upon request by any qualified investigator.
