# Supplementary material for: Pupillary responses to the glare illusion in normal pressure hydrocephalus: insights into network dysfunction and neurodegenerative comorbidities
Source: Neurol Sci. 2026 Apr 9;47(5):408. doi: 10.1007/s10072-026-09015-2 (PMC13061757; doi:10.1007/s10072-026-09015-2)
Supplement: Supplementary file 1 — Supplementary file1 (DOCX 17 KB) [file 10072_2026_9015_MOESM1_ESM.docx]

**Supplementary Table 1. Exploratory analysis of the Illusion Effect Index (IEI) between healthy controls and NPH**

|  | HC (n=38) | NPH (n=43) | U | p |
| --- | --- | --- | --- | --- |
| IEI constriction amplitude | −0.020 [−0.075–0.015] | −0.023 [−0.071–0.003] | 778.000 | 0.717 |
| IEI constriction velocity | −7.94×10⁻⁵ [−4.01×10⁻⁴–1.20×10⁻⁴] | −5.45×10⁻⁵ [−2.65×10⁻⁴–6.16×10⁻⁵] | 817.000 | 1.000 |

Data are presented as median [interquartile range].

Group comparisons were performed using the Mann–Whitney U test, as the data were not normally distributed.

No significant differences were observed between healthy controls and patients with normal pressure hydrocephalus.

IEI = Illusion Effect Index, calculated as the difference between pupillary responses to glare and control stimuli (Glare − Control)

HC = healthy controls, NPH = normal pressure hydrocephalus
