# Supplementary material for: Pupillary responses to the glare illusion in normal pressure hydrocephalus: insights into network dysfunction and neurodegenerative comorbidities
Source: Neurol Sci. 2026 Apr 9;47(5):408. doi: 10.1007/s10072-026-09015-2 (PMC13061757; doi:10.1007/s10072-026-09015-2)
Supplement: Supplementary file 2 — Supplementary file2 (DOCX 17 KB) [file 10072_2026_9015_MOESM2_ESM.docx]

**Supplementary Table 2. Exploratory analysis of the Illusion Effect Index (IEI) between Normal-DAT and Low-DAT groups**

|  | Normal-DAT (n=31) | Low-DAT (n=11) | U | p |
| --- | --- | --- | --- | --- |
| IEI Constriction amplitude | −0.027 [−0.073–0.003] | −0.020 [−0.038–0.004] | 195.000 | 0.498 |
| IEI Constriction velocity | −9.61×10⁻⁵ [−2.61×10⁻⁴–1.35×10⁻⁵] | 6.19×10⁻⁵ [−1.71×10⁻⁴–1.21×10⁻⁴] | 217.000 | 0.191 |

Data are presented as median [interquartile range].

Group comparisons were performed using the Mann–Whitney U test, as the data were not normally distributed..

No significant differences were observed between Normal-DAT and Low-DAT groups.

IEI = Illusion Effect Index, calculated as the difference between pupillary responses to glare and control stimuli (Glare − Control)

DAT = dopamine transporter, Normal-DAT = NPH patients with normal DAT uptake, Low-DAT = NPH patients with reduced DAT uptake
